# Supplementary material for: Overview of the Cast Polyolefin Film Extrusion Technology for Multi-Layer Packaging Applications
Source: Materials (Basel). 2023 Jan 26;16(3):1071. doi: 10.3390/ma16031071 (PMC9920539; doi:10.3390/ma16031071)
Supplement: Supplementary file 1 [file materials-16-01071-s001.zip › materials-2156546-supplementary.pdf]

materials

MDPI

Supplementary Materials

Overview of the Cast Polyolefin Film Extrusion Technology for Multi-Layer Packaging Applications

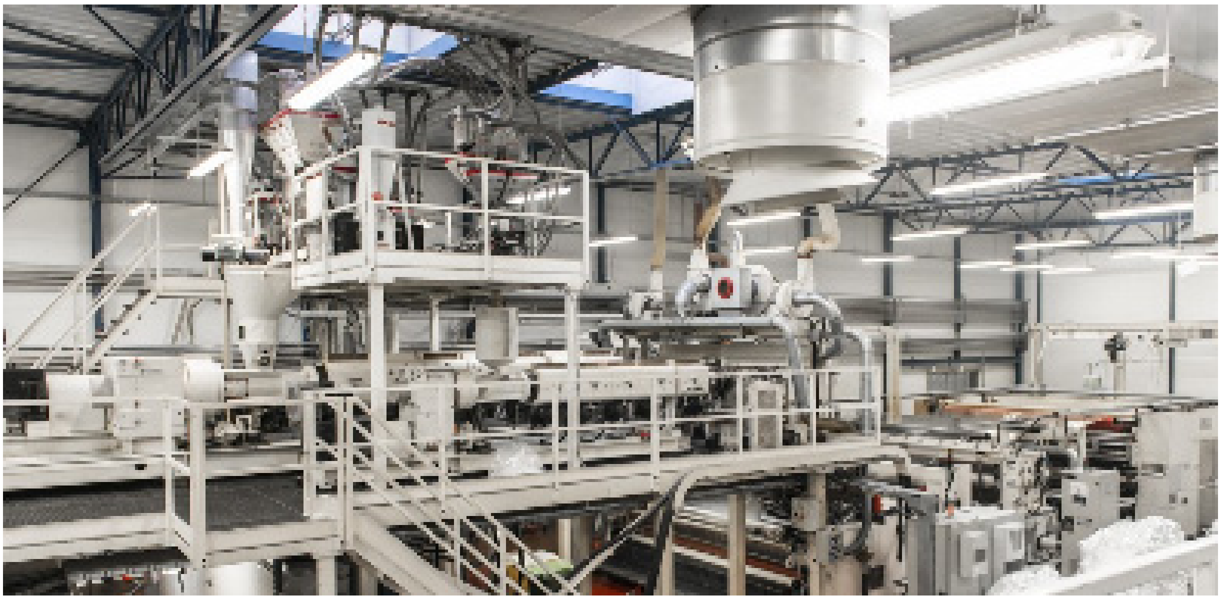

Figure S1. The industrial line for extrusion of a three-layer cast polypropylene film.

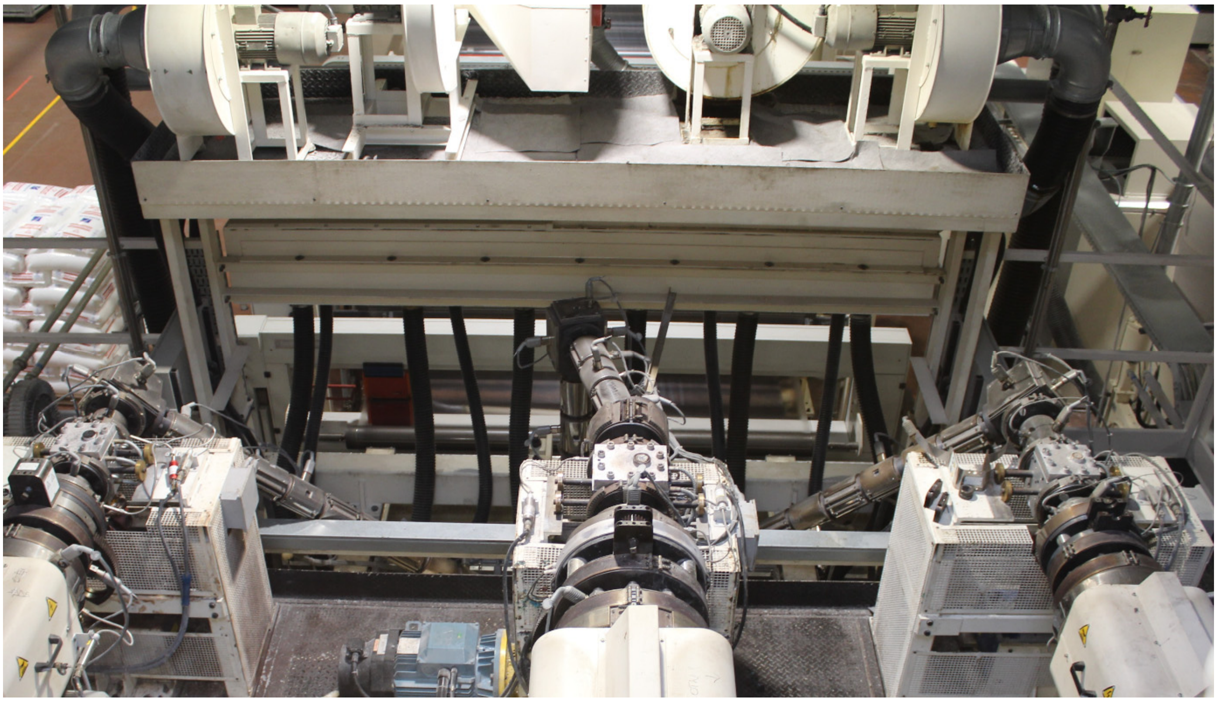

Figure S2. Arrangement of extruders in line for extrusion of a cast three-layer pol-propylene film.

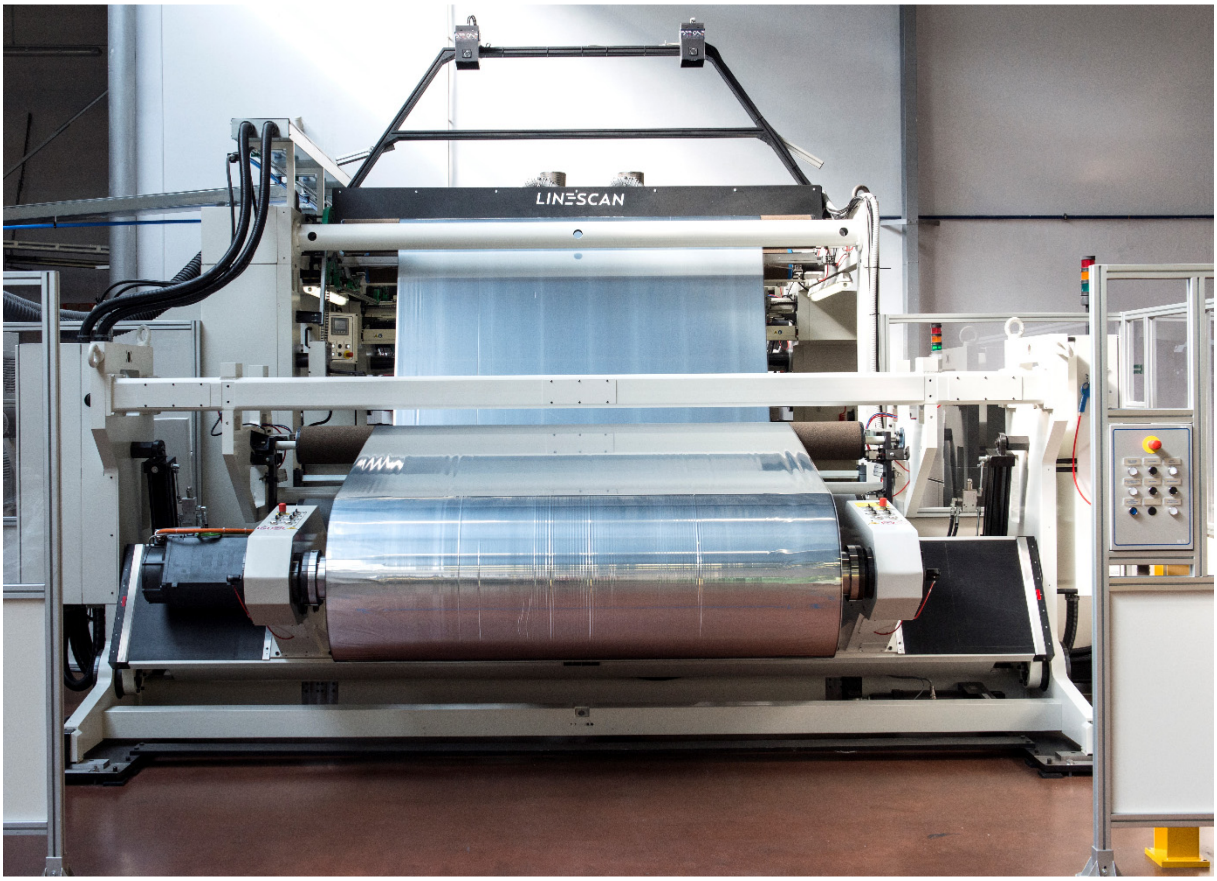

Figure S3. The system of receiving rollers with the winder.
